# Risk factors for poor sleep quality in patients with inflammatory bowel disease in China: A multicenter study

**DOI:** 10.3389/fpsyt.2023.1130396

**Published:** 2023-03-09

**Authors:** Suqi Zeng, Chuan Liu, Jixiang Zhang, Ping An, Zhongchun Liu, Changqing Jiang, Jie Shi, Kaichun Wu, Weiguo Dong

**Affiliations:** ^1^Department of Gastroenterology, Renmin Hospital of Wuhan University, Wuhan, China; ^2^Department of Psychiatry, Renmin Hospital of Wuhan University, Wuhan, China; ^3^Department of Clinical Psychology, Beijing Anding Hospital, Capital Medical University, Beijing, China; ^4^Department of Medical Psychology, Chinese People’s Liberation Army Rocket Army Characteristic Medical Center, Beijing, China; ^5^Department of Gastroenterology, Xijing Hospital, Air Force Medical University, Xi’an, China

**Keywords:** inflammatory bowel disease, psychological symptoms, sleep quality, nomogram, hurdle model

## Abstract

**Objectives:**

This study aimed to determine the prevalence and risk factors for poor sleep quality in inflammatory bowel disease (IBD) patients.

**Methods:**

2,478 IBD patients were enrolled to investigate their sleep quality using the Pittsburgh sleep quality index (PSQI). Clinical and psychological characteristics were collected to explore the risk factors for poor sleep quality. A hurdle model was conducted to predict poor sleep quality based on the risk factors. Among this hurdle model, the logistic regression model was used to determine risk factors of the presence of poor sleep quality, and the zero-inflated negative binomial model was employed to identify risk factors of the severity of poor sleep quality.

**Results:**

In this study, 1,491 (60.17%) IBD patients had poor sleep quality, and the proportion in the older group was higher than younger group (64.89% vs. 58.27%, *p* = 0.003). According to multivariable logistic regression, age (OR, 1.011; 95% CI [1.002,1.020]; *p* = 0.014), Patient Health Questionnaire-9 (PHQ-9) score (OR, 1.263; 95% CI [1.228,1.300]; *p* < 0.001), systemic (OR, 0.906; 95% CI [0.867,0.946]; *p* < 0.001) and emotional performance (OR, 1.023; 95% CI [1.005,1.043]; *p* = 0.015) were risk factors of the presence of poor sleep quality. The area under the curve (AUC) of the prediction model was 0.808. According to zero-truncated negative binomial regression, age (RR, 1.004; 95% CI [1.002,1.005]; *p* < 0.001) and PHQ-9 score (RR, 1.027; 95% CI [1.021,1.032]; *p* < 0.001) were risk factors of the severity of poor sleep quality.

**Conclusion:**

The prevalence of poor sleep quality among the older group in IBD patients was relatively high. Old age and depressive mood are risk factors for both the presence and severity of poor sleep quality.

## 1. Introduction

Inflammatory bowel disease (IBD), represented by Crohn’s disease (CD) and ulcerative colitis (UC), are chronic and debilitating disorders waning with a relapsing–remitting course (1). Besides physiological discomfort, IBD patients frequently suffer from a high level of psychological distress and low quality of life (2). 25 and 32% of IBD patients have signs of depression and anxiety, respectively (3). Poor sleep quality is highly prevalent in active IBD patients, ranging from 73 to 100% (4), much higher than in healthy controls. Inactive IBD patients were also found to have poorer sleep quality than healthy controls, suggesting that sleep quality in IBD patients is not only due to IBD-related symptoms (5). Therefore, it needs to describe the associations between sleep quality and IBD to inform the development of therapies that enhance the quality of life among IBD patients.

Sleep is an essential and intricate physiological process for maintaining physiological and psychological health. Physiologically, sleep regulates the immune and neuroendocrine systems, and it has been investigated as a possible marker of subclinical inflammation (6). Clinical studies have shown that poor sleep quality may result in exacerbations in IBD patients (7). Psychologically, poor sleep quality can worsen depression and fatigue symptoms, impair social functioning, and impact the quality of life (8). Therefore, there is a complex and bidirectional relationship between poor sleep quality and immune-mediated disease: poor sleep quality can lead to a reduction in quality of life and fatigue; it might also exacerbate symptoms such as pain and discomfort, which in turn affect sleep quality (9). It also found that poor sleep quality is one of the main concerns for patients with chronic intestinal disease, which can negatively impact health and work productivity in the long term (6). As an immune-related gastrointestinal disease, the sleep quality in IBD can affect not only its symptoms but also might act as an extraintestinal manifestation and aggravating or etiological factor (5). However, although poor sleep quality is a significant health concern among IBD patients, it is often underappreciated in clinical medicine.

Ageing is also linked to poor sleep quality, including reduced sleep duration, decreased sleep efficiency, and decreased quantity of slow wave and rapid eye movement (REM) sleep (10). However, poor sleep quality in the elderly is not always caused by ageing alone. Changes in physical or mental health and social involvement, lifestyle, and surroundings are all related to the ageing process and can negatively impact sleep quality. A recent meta-analysis showed that the prevalence of poor sleep quality increased with age (11). However, clinical and psychological characteristics between older and younger IBD patients have been compared in few previous studies. Due to the underrepresentation of elderly patients in clinical trials and the absence of particular treatment algorithms, their management has been inadequately studied (12), preventing future investigation into the precise causes of their poor sleep quality. With the ageing population, the non-standard treatment of elderly IBD patients needs to be changed urgently based on the investigation of their contemporary clinical and psychological characteristics.

The causes of poor sleep quality in IBD patients were not fully understood, but a growing number of recent studies showed that sleep quality might be a previously underestimated risk factor for adverse outcomes (13). Symptom control may improve sleep quality to some extent, but prolonged periods of poor sleep quality can result in conditioned insomnia, which may require multidisciplinary treatment (7). Therefore, it is essential to evaluate the sleep quality of IBD patients timely and take appropriate improvement measures. It is not practical for all IBD patients to specifically evaluate subjective or objective sleep quality, so it is feasible to predict the sleep quality of IBD patients through readily available clinical data. Few previous studies have investigated the factors associated with sleep quality in IBD patients, but these studies have always focused on assessing the presence of poor sleep quality without further exploring the factors associated with the severity of poor sleep quality (1, 8). Moreover, previous studies only remained at finding related factors and did not develop practical clinical tools to predict the sleep quality of IBD patients rapidly. Therefore, based on exploring predictive factors for the presence and severity of poor sleep quality in IBD patients, we created a clinical prediction model that can effectively predict the presence and severity of poor sleep quality in IBD patients, so as to help clinicians quickly evaluate the sleep quality of IBD patients.

## 2. Participants and methods

### 2.1. Study design and participants

This national multicenter cross-sectional study was conducted by the Psychology Club of IBD Group of Gastroenterology Society of Chinese Medical Association and the Chinese Association for Mental Hygiene between September 2021 and May 2022. Sixty-six gastroenterologists from 42 hospitals in 22 provinces (autonomous regions and municipalities directly under the central government) participated in this study, and a total of 2,478 valid questionnaires were collected. The questionnaires collected information on sociodemographic characteristics, disease activity (judged according to the “consensus opinion on the diagnosis and treatment of IBD” (14)), illness duration, symptoms (e.g., diarrhea, abdominal pain, hematochezia, extraintestinal manifestations, and complications), and prior therapies in IBD patients. According to the “consensus opinion on the diagnosis and treatment of IBD” (14), the modified Mayo score was used to evaluate the disease activity of UC patients (≤2 as remission, >2 as active), and the CDAI score was used to assess the disease activity of CD patients (<150 as remission, ≥150 as active). Meanwhile, various scales were used to assess patients’ mental health and quality of life. Following were the eligibility requirements: (1) age ≥ 18 years old, (2) diagnosed with IBD (14), (3) willing to be surveyed (e.g., undergo physician management and psychological investigation, obey the administration of medication compliance), (4) information was complete. Patients were excluded from the study if they had a previous history of mental illness or combined with other diseases that seriously affect the quality of life. Ultimately, 1,107 CD patients and 1,371 UC patients were included (Supplementary Figure 1). Based on the definition of “middle age” in Merriam-Webster (15) and the Oxford Learner’s Dictionary (16), we divided the 2,478 IBD patients into the following age groups according to their legal age: 937 CD and 829 UC patients (all aged<45 years) were divided into the younger group, and 170 CD and 542 UC patients (all aged≥45 years) were divided into the older group. To develop and validate the models relating to sleep quality, patients from the 42 centers were randomly divided into training (*n* = 1735[70.02%]) and validation (*n* = 743[29.98%]) cohorts.

### 2.2. Assessment of sleep quality, psychological symptoms, and quality of life

Each participant self-assessed their sleep quality within the preceding several months through the Pittsburgh Sleep Quality Index (PSQI) (17), a standardized, self-administered questionnaire covering seven subscales of sleep quality (sleep quality, sleep latency, sleep duration, sleep efficiency, sleep disturbance, sleep medication, and daily dysfunction). The Chinese version of the PSQI has been demonstrated to be reliable (Cronbach’s α: 0.84) and valid (factor loading of each component:>0.5) among Chinese students (18). A PSQI score more excellent than five indicates poor sleep quality.

This study utilized the Patient Health Questionnaire (PHQ-9), the Generalized Anxiety Disorder-7(GAD-7), and the Inflammatory Bowel Disease Questionnaire (IBDQ) to assess psychological symptoms and quality of life. The Chinese version of the above questionnaires used in this study have all been verified to have great reliability and validity (19–21). The PHQ-9 contains nine items, each evaluated from 0 to 3. The GAD-7 has seven questions, each assigned a score between 0 to 3. No symptoms (1–4), mild (5–9), moderate (10–15), and severe (15+) are the score ranges for the severity of anxiety and depression symptoms on the PHQ-9 and GAD-7. The CFF/ECFS guideline recommends supportive interventions and rescreening for individuals with mild symptoms. Following the degree CFF/ECFS guidelines recommend psychological/psychopharmacological interventions, a cut-off score of “10+” was employed in the study to identify depression and anxiety (22). The IBDQ score based on responses to questions concerning bowel habits and social, systemic, and emotional performance ranges from 32 to 224. A higher score indicates a better quality of life.

### 2.3. Statistical analysis

The results of descriptive analyses were presented in terms of absolute counts (*n*) and relative frequencies (%) for categorical variables, along with the mean ± standard deviation ($\bar{x}$±*s*) for continuous variables. The Mann–Whitney U, Chi-squared, and Fisher’s exact tests were used to examine differences between age groups. Multivariate logistic regression analysis was further performed to calculate the adjusted odds ratios (ORs) and 95% confidence intervals for the significant independent variables between age groups.

We calculated the sample size required for the questionnaire survey according to the international principles of psychometrics and questionnaire design. If the number of items in the questionnaire rises, the recommended sample size will be increased, which is 5–20 times the number of items in the questionnaire. If N is the number of questionnaire items in this research, the sample size needs to be at least 5 *N*. In addition, about 10% of the questionnaires may be withdrawn or invalid from the survey, so the sample size needs to be 5.5 *N*. Consequently, the sample size should be more than 451. Additionally, we calculated the required sample size for conducting the prediction model according to the formula developed by Riley et al. (23) and the “pmsampsize” package (R 4.1.0). Combined with the study conducted by Leal et al. (24), which showed the proportion of poor sleep quality in IBD patients was 44.9%, the arguments set as follow: rsquared = 0.34, parameters = 10, prevalence = 0.449. The obtaining required sample size was 381, and the events per candidate predictor parameter was 17.11. Considering China’s large population and other confounding factors, we tried to expand the sample size, 2,478 questionnaires were finally included in the study. And the final included questionnaires to develop the prediction model was 1735, which was much larger than the required sample size.

For conducting the hurdle model, all patients were randomly divided into a training (70%) and a validation (30%) cohort. The hurdle model was comprised of two sub-models (the logistic regression model and the zero-inflated negative binomial model) that examined two outcomes: the presence and severity of poor sleep quality. The training cohort was used to derive the two sub-models, and the validation cohort was used to validate the logistic regression model. Variables with *p*-values < 0.05 from univariate analyses in the training cohort were incorporated into the hurdle model. Variables selection in logistic regression was performed using a backward stepwise method. Based on the selected variables, a nomogram was established. The nomogram’s discriminative ability, calibration and net clinical benefit were assessed using the area under the receiver operating characteristics (AUC), calibration plot and decision curve in the training and validation cohort, respectively. After converting PSQI less than or equal to five to zero, we conducted the zero-inflated negative binomial model. For each predictor, odds ratio (for the logistic regression sub-model), risk ratios (for the zero-truncated negative binomial regression sub-model), and associated 95% confidence intervals were calculated.

All statistical analyses were performed using R 4.1.0 statistics software (R Foundation for Statistical Computing, Vienna, Austria) and Origin 2022 software.

## 3. Results

### 3.1. Age-related differences

#### 3.1.1. Patient characteristics

2,478 patients were enrolled, including 1,107 CD patients (44.67%) and 1,371 UC patients (55.33%). The younger group comprised 937 CD and 829 UC patients, and the older group included 170 CD and 542 UC patients.

**Figure 1:**
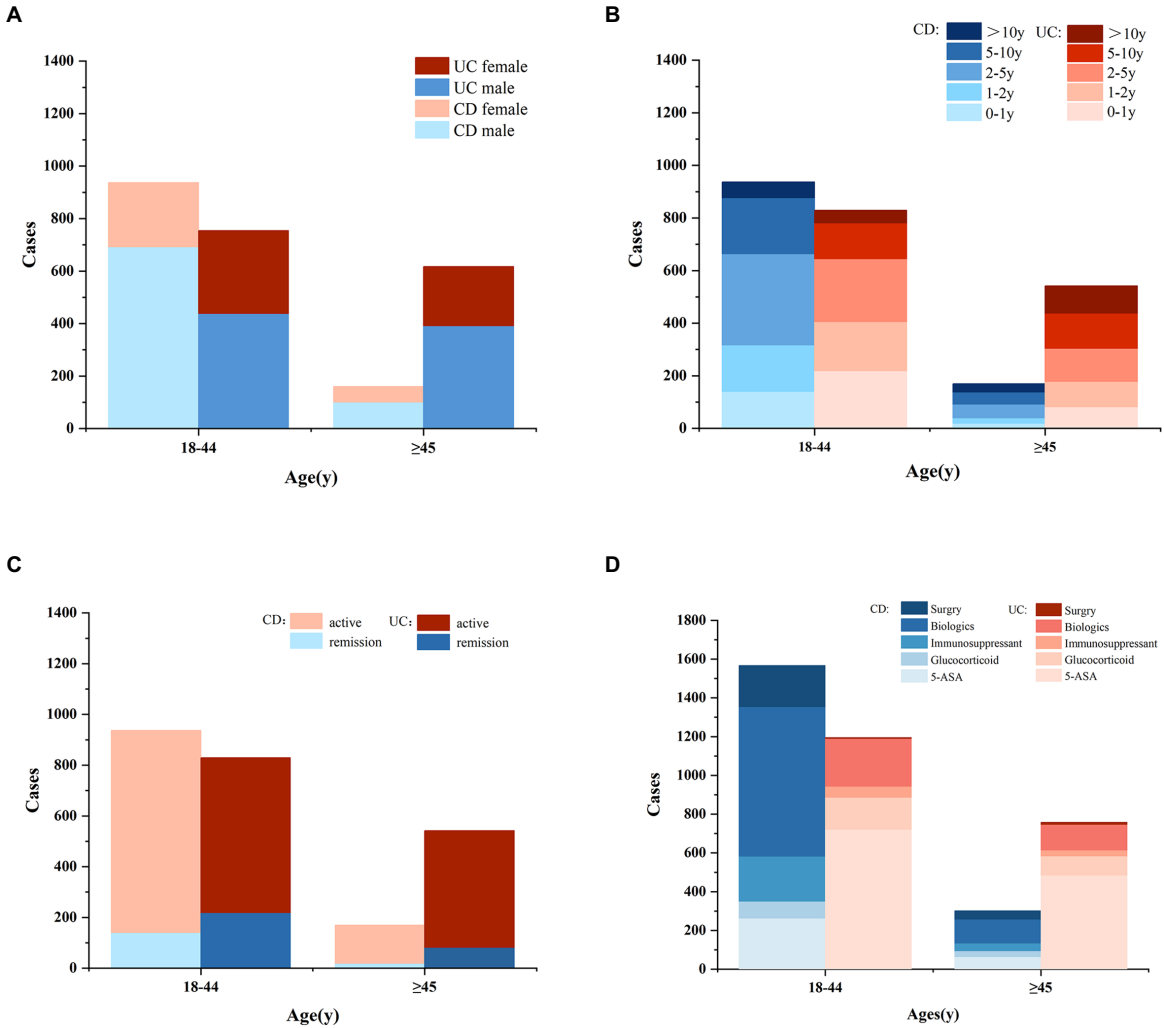
The cases of gender **(A)**, illness duration **(B)**, disease activity **(C)**, and prior therapy **(D)** in IBD patients of different age groups (CD, Crohn’s disease; UC, ulcerative colitis; ASA, amino salicylic acid).

Table 1 and Figure 1 have presented the sociodemographic characteristics, clinical characteristics, symptoms, and prior therapies of patients in different age groups. Among CD patients of different age groups, there were significant differences in gender, illness duration, usage of 5-Amino salicylic acid (5-ASA), glucocorticoids, and biologics (all *p* ≤ 0.05), but not in weight, disease activity, symptoms, or the proportion of the first visit. Among UC patients of different age groups, illness duration, usage of biologics, and surgical history differed significantly (all *p* ≤ 0.05), while gender, disease activity, symptoms, and other prior therapies did not.

**Table 1:** Sociodemographic characteristics, clinical characteristics, symptoms, and prior therapy in IBD patients of different age groups.

		CD			UC	
		*N* = 1,107(44.67%)			*N* = 1,371(55.33%)	
	18–44 years	≥45 years	*p*	18–44 years	≥45 years	*p*
	*N* = 937(84.64%)	*N* = 170(15.36%)		*N* = 829(60.47%)	*N* = 542(39.53%)	
Sociodemographic characteristics						
Age (years)	29.78 ± 6.82	53.34 ± 6.69	<0.001	33.11 ± 6.41	54.69 ± 7.51	<0.001
Sex (male)	692(73.85%)	101(59.41%)	<0.001	438(52.83%)	316(71.49%)	0.0531
Weight (kg)	60.26 ± 11.61	59.81 ± 11.43	>0.999	61.77 ± 11.94	63.80 ± 11.51	<0.001
First visit	125(13.34%)	25(14.71%)	0.721	207(24.97%)	136(30.77%)	>0.999
Clinical characteristics						
Illness duration (years)			<0.001			<0.001
<1	140(14.84%)	19(11.18%)		219(26.42%)	82(18.55%)	
1–2	177(18.89%)	21(12.35%)		187(22.56%)	96(17.71%)	
>2–5	347(37.03%)	52(30.59%)		239(28.83%)	126(23.25%)	
>5–10	213(22.73%)	46(27.06%)		136(16.41%)	134(24.72%)	
>10	60(6.40%)	32(18.82%)		48(5.79%)	104(19.19%)	
Disease activity			0.360			0.261
Remission	467(49.84%)	75(44.12%)		235(28.35%)	169(31.18%)	
Active	470(50.16%)	95(55.88%)		594(71.65%)	373(68.82%)	
Symptoms						
Diarrhea	497(53.04%)	93(54.71%)	0.752	548(66.10%)	379(69.93%)	0.156
Hematochezia	157(16.76%)	38(22.35%)	0.098	536(64.66%)	327(60.33%)	0.118
Abdominal pain	609(64.99%)	113(66.47%)	0.776	438(52.83%)	259(47.79%)	0.076
Systemic complications	63(6.72%)	5(2.94%)	0.086	19(2.29%)	21(3.87%)	0.124
Extraintestinal manifestations	93(9.93%)	9(5.29%)	0.352	58(7.00%)	38(7.01%)	>0.999
Complications	155(16.54%)	18(10.59%)	0.064	15(1.81%)	17(3.14%)	0.159
Prior therapies						
5-ASA	262(27.96%)	63(37.06%)	0.021	721(86.97%)	484(89.30%)	0.228
Glucocorticoid	88(9.39%)	31(18.24%)	0.001	165(19.90%)	99(18.27%)	0.495
Immunosuppressants	232(24.76%)	40(23.53%)	0.806	57(6.88%)	31(5.72%)	0.459
Biologics	771(82.28%)	123(72.35%)	0.004	246(29.67%)	132(24.35%)	0.036
Surgery	214(22.84%)	45(26.47%)	0.352	6(0.72%)	12(2.21%)	0.033

CD=Crohn’s disease; UC = ulcerative colitis; IBD = inflammatory bowel disease; ASA = amino salicylic acid.

#### 3.1.2. Sleep quality, psychological symptoms, and quality of life

In this study, 1,491 (60.17%) IBD patients had poor sleep quality. As shown in Table 2 and Supplementary Figure 2, the PSQI score was the only mental-related characteristic that differed significantly between age groups in both CD and UC patients (*p* < 0.01). Both CD and UC patients showed higher PSQI scores in the older group, and there was no significant difference in PSQI scores between CD and UC patients (*p* > 0.05; Supplementary Table 1; Supplementary Figure 2), indicating that older IBD patients have poorer sleep quality. Additionally, as indicated in Supplementary Table 1, the PHQ-9 and GAD-7 scores did not significantly differ between CD and UC. Therefore, these two groups’ PSQI, PHQ-9, and GAD-7 scores were merged for analysis. According to Supplementary Table 2, there was no significant difference in the proportion of IBD patients who had symptoms of depression and anxiety across age groups (*p* > 0.05). However, poor sleep quality was present in 64.89% of older patients with IBD, and 58.27% of younger patients with IBD, showing a significant difference between age groups (*p* < 0.01). CD patients’ bowel habits and social performance were likewise considerably worsened in the older group (*p* < 0.01), whereas UC patients did not exhibit such differences (*p* > 0.05; Table 2; Supplementary Figure 3). As shown in Supplementary Table 1 and Supplementary Figure 3, CD patients received significantly higher IBDQ scores than UC patients in terms of scores for bowel habits, systemic performance, emotional performance, and social performance (*p* < 0.05), indicating that CD patients had a superior quality of life than UC patients.

**Table 2:** PSQI, PHQ-9, GAD-7 and IBDQ scores in IBD patients of different age groups.

		CD			UC	
		*N* = 1,107(44.67%)			*N* = 1,371(55.33%)	
	18–44 years	≥45 years	*p*	18–44 years	≥45 years	*p*
	*N* = 937(84.64%)	*N* = 170(15.4%)		*N* = 829(60.50%)	*N* = 542(39.5%)	
PSQI	6.61 ± 3.60	7.68 ± 4.15	0.002	6.67 ± 3.58	7.51 ± 4.08	<0.001
PHQ-9	6.98 ± 5.89	7.20 ± 6.00	0.677	7.13 ± 6.31	6.93 ± 6.18	0.651
GAD-7	6.31 ± 4.83	6.55 ± 5.43	0.932	7.01 ± 5.38	6.66 ± 5.60	0.089
IBDQ	180.70 ± 28.05	176.14 ± 30.07	0.064	172.58 ± 32.08	172.23 ± 33.34	0.991
Bowel habits	59.94 ± 8.82	57.93 ± 9.49	0.009	56.16 ± 10.72	55.55 ± 11.00	0.332
Systemic performance	27.55 ± 5.13	26.85 ± 5.21	0.095	26.84 ± 5.42	26.48 ± 5.84	0.375
Emotional performance	67.65 ± 12.06	66.54 ± 12.59	0.313	64.66 ± 13.04	65.11 ± 13.30	0.431
Social performance	25.56 ± 4.75	24.83 ± 4.88	0.020	24.92 ± 5.11	25.09 ± 5.37	0.440

CD=Crohn’s disease; UC = ulcerative colitis; PSQI=Pittsburgh Sleep Quality Index; PHQ-9 = Patient Health Questionnaire-9; GAD-7 = Generalized Anxiety Disorder 7-item Scale; IBDQ = Inflammatory Bowel Disease Quality-of-Life Questionnaire.

#### 3.1.3. Independent factors associated with age

According to Supplementary Figure 4A, gender, illness duration, the usage of glucocorticoids or biologics, and the PSQI score were the independent factors associated with age in CD patients. And in UC patients, weight, illness duration, the usage of biologics, and the PSQI score were independent factors associated with age (Supplementary Figure 4B). Noting that the PSQI score was independently associated with age in IBD patients, we conducted further studies to investigate age’s impacts on sleep quality and predictors of poor sleep quality.

### 3.2. Predicting the presence and severity of poor sleep quality

#### 3.2.1. Patient characteristics

A training cohort (1735 cases) and a validation cohort (743 cases) were created randomly using all patients’ data, with a ratio of 7:3. The sociodemographic characteristics, clinical characteristics, symptoms, and prior therapies of patients in the training and validation cohorts were shown in Table 3. The training and validation cohort data partition appeared to be balanced based on the *p*-values (*p* > 0.05).

**Table 3:** Sociodemographic characteristics, clinical characteristics, symptoms, and prior therapies of patients in the training and validation cohorts.

Characteristic	Whole population *N* = 2,478	Training cohort *N* = 1735(70.02%)	Validation cohort *N* = 743(29.98%)	*p*
Sociodemographic characteristics				
Age (years)	37.96 ± 12.54	37.99 ± 12.58	37.89 ± 12.47	0.919
Sex (male)	1,547(62.43%)	1,094(63.05%)	453(60.97%)	0.349
Weight (kg)	61.51 ± 11.76	61.74 ± 11.85	60.98 ± 11.54	0.096
First visit	493(19.90%)	327(18.85%)	166(22.34%)	0.052
Category				0.358
CD	1,107(44.67%)	786(45.30%)	321(43.20%)	
UC	1,371(55.33%)	949(54.70%)	422(56.80%)	
Clinical characteristics				
Illness duration (years)				0.186
<1	460(18.56%)	304(17.52%)	156(21.00%)	
1–2	481(19.41%)	341(19.65%)	140(18.84%)	
>2–5	764(30.83%)	529(30.49%)	235(31.63%)	
>5–10	529(21.35%)	384(22.13%)	145(19.52%)	
>10	244(9.85%)	177(10.20%)	67(9.02%)	
Disease activity				0.122
Remission	946(38.18%)	680(39.19%)	266(35.80%)	
Active	1,532(61.82%)	1,055(60.81%)	477(64.20%)	
Symptoms				
Diarrhea	1,517(61.22%)	1,059(61.04%)	458(61.64%)	0.812
Hematochezia	1,058(42.70%)	727(41.90%)	331(44.55%)	0.239
Abdominal pain	1,419(57.26%)	996(57.41%)	423(56.93%)	0.861
Systemic complications	108(4.36%)	80(4.61%)	28(3.77%)	0.404
Extraintestinal manifestations	198(7.99%)	138(7.95%)	60(8.08%)	0.983
Complications	205(8.27%)	143(8.24%)	62(8.34%)	0.996
Prior therapy				
5-ASA	1,530(61.74%)	1,060(61.10%)	470(63.26%)	0.332
Glucocorticoid	383(15.46%)	249(14.35%)	134(18.04%)	0.024
Immunosuppressants	360(14.53%)	246(14.18%)	114(15.34%)	0.489
Biologics	1,272(51.33%)	913(52.62%)	359(48.32%)	0.055
Psychological symptoms				
PHQ-9	7.04 ± 6.10	7.04 ± 6.09	7.03 ± 6.13	0.969
GAD-7	6.64 ± 5.24	6.65 ± 5.20	6.61 ± 5.33	0.716
Quality of life				
IBDQ	175.80 ± 31.00	176.36 ± 31.16	174.55 ± 30.62	0.116
Bowel habits	57.58 ± 10.20	57.74 ± 10.28	57.20 ± 10.00	0.092
Systemic performance	27.03 ± 5.41	27.12 ± 5.44	26.81 ± 5.35	0.133
Emotion performance	66.02 ± 12.77	66.22 ± 12.74	65.54 ± 12.84	0.214
Social performance	25.19 ± 5.03	25.27 ± 5.03	25.01 ± 5.01	0.198

#### 3.2.2. Predictors for the presence of poor sleep quality

As shown in Table 4, poor sleep quality was related to age, gender, weight, illness duration, disease activity, PHQ-9, GAD-7, usage of biologics and the IBDQ score (including bowel habits, systemic performance, emotional performance, and social performance) in the univariate analysis (*p* < 0.05). According to Table 5, using a backward stepwise selection method with the AIC in logistic regression modeling, the following four factors were found to be the predictors of poor sleep quality: age (OR, 1.011; 95% CI [1.002,1.020]; *p* = 0.014), PHQ-9 (OR, 1.263; 95% CI [1.228,1.300]; *p* < 0.001), systemic performance (OR, 0.906; 95% CI [0.867,0.946]; *p* < 0.001)and emotional performance (OR, 1.023; 95% CI [1.005,1.043]; *p* = 0.015).

**Table 4:** Univariate analysis of variables for poor sleep quality in the training cohort.

	Poor Sleep Quality	Good Sleep Quality		
	PSQI>5	PSQI≤5		
Factors	*N* = 1,030(59.37%)	*N* = 705(40.63%)	OR (95%CI)	*p*
Age (years)	38.54 ± 12.75	37.20 ± 12.30	1.009(1.001,1.016)	0.030
Category				
CD	459(44.56%)	327(46.38%)	1	
UC	571(55.44%)	378(53.62%)	1.076(0.888,1.304)	0.455
Gender				
Male	623(60.49%)	471(66.81%)	1	
Female	407(39.51%)	234(33.19%)	1.315(1.077, 1.608)	0.007
First visit	208(20.19%)	119(16.88%)	1.246(0.973,1.602)	0.083
Weight (kg)	61.22 ± 11.99	62.49 ± 11.61	0.991(0.983,0.999)	0.029
Illness duration (years)				
<1	174(16.89%)	130(18.44%)	1.000	
1–2	181(17.57%)	160(22.70%)	0.845(0.619,1.153)	0.289
>2–5	325(31.55%)	204(28.94%)	1.190(0.893,1.585)	0.234
>5–10	251(24.37%)	133(18.87%)	1.410(1.035,1.923)	0.030
>10	99(9.61%)	78(11.06%)	0.948(0.653,1.379)	0.781
Disease activity				
Remission	380(36.89%)	300(42.55%)	1.000	
Active	650(63.11%)	405(57.45%)	1.267(1.042,1.541)	0.018
Symptoms				
Diarrhea	633(61.46%)	426(60.43%)	1.044(0.858,1.271)	0.665
Hematochezia	443(43.01%)	284 (40.28%)	1.119(0.921,1.360)	0.258
Abdominal pain	593(57.57%)	403(57.16%)	1.017(0.838,1.234)	0.865
Systemic complications	51(4.95%)	29(4.11%)	1.214(0.767,1.957)	0.414
Extraintestinal manifestations	87(8.45%)	51(7.23%)	1.183(0.829,1.705)	0.360
Complications	80(7.77%)	63(8.94%)	0.858(0.609,1.215)	0.385
Prior therapy				
5-ASA	638(61.94%)	422(59.86%)	1.091(0.897,1.328)	0.382
Glucocorticoid	146(14.17%)	103(14.61%)	0.965(0.736,1.270)	0.800
Immunosuppressants	143(13.88%)	103(14.61%)	0.942(0.718,1.241)	0.670
Biologics	521(50.58%)	392(55.60%)	0.817(0.674,0.990)	0.040
Surgery	117(11.36%)	92(13.05%)	0.854(0.638,1.145)	0.288
Psychological symptoms				
PHQ-9	9.42 ± 6.24	3.55 ± 3.74	1.277((1.243,1.313)	<0.001
GAD-7	8.42 ± 5.22	4.06 ± 3.93	1.232(1.201,1.265)	<0.001
Quality of life				
IBDQ	169.94 ± 31.85	185.74 ± 27.58	0.982(0.979,0.986)	<0.001
Bowel habits	56.01 ± 10.70	60.27 ± 9.07	0.957(0.947,0.967)	<0.001
Systemic performance	25.88 ± 5.58	28.94 ± 4.66	0.890(0.872,0.908)	<0.001
Emotion performance	63.57 ± 13.01	70.10 ± 11.28	0.957(0.948,0.965)	<0.001
Social performance	24.47 ± 5.15	26.44 ± 4.61	0.921(0.902,0.940)	<0.001

CD = Crohn’s disease; UC = ulcerative colitis; PSQI = Pittsburgh Sleep Quality Index; PHQ-9 = Patient Health Questionnaire-9; GAD-7 = Generalized Anxiety Disorder 7-item Scale; IBDQ = Inflammatory Bowel Disease Quality-of-Life Questionnaire; OR = odds ratio; CI = confidence interval.

**Table 5:** Risk factors of the presence of poor sleep quality derived from backward stepwise logistic regression analysis.

Characteristics	B (SE)	OR (95%CI)	*p*-value
Age	0.011 (0.005)	1.011(1.002,1.020)	0.014
Gender	0.201 (0.120)	1.223(0.967,1.547)	0.094
PHQ-9	0.234 (0.146)	1.263(1.228,1.300)	<0.001
Systemic performance	−0.099 (0.022)	0.906(0.867,0.946)	<0.001
Emotional performance	0.023 (0.009)	1.023(1.005,1.043)	0.015

PHQ-9 = Patient Health Questionnaire-9; OR = odds ratio; CI = confidence interval.

#### 3.2.3. Development and validation of the nomogram

**Figure 2:**
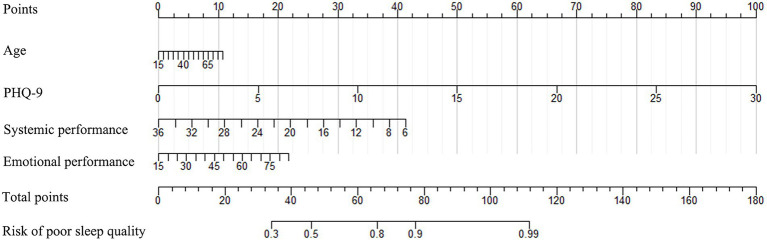
The nomogram used to predict poor sleep quality in IBD patients (PHQ-9, Patient Health Questionnaire-9).

**Figure 3:**
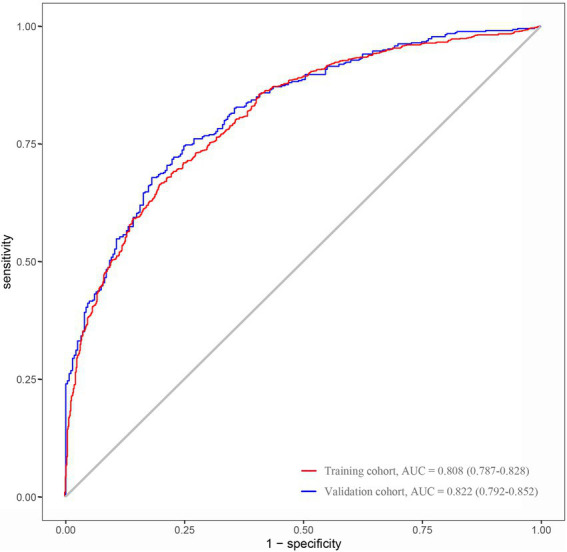
AUCs of the nomogram used to predict the presence of poor sleep quality in the training and validation cohorts (ROC, receiver operating characteristic; AUC, the area under the ROC curve).

**Figure 4:**
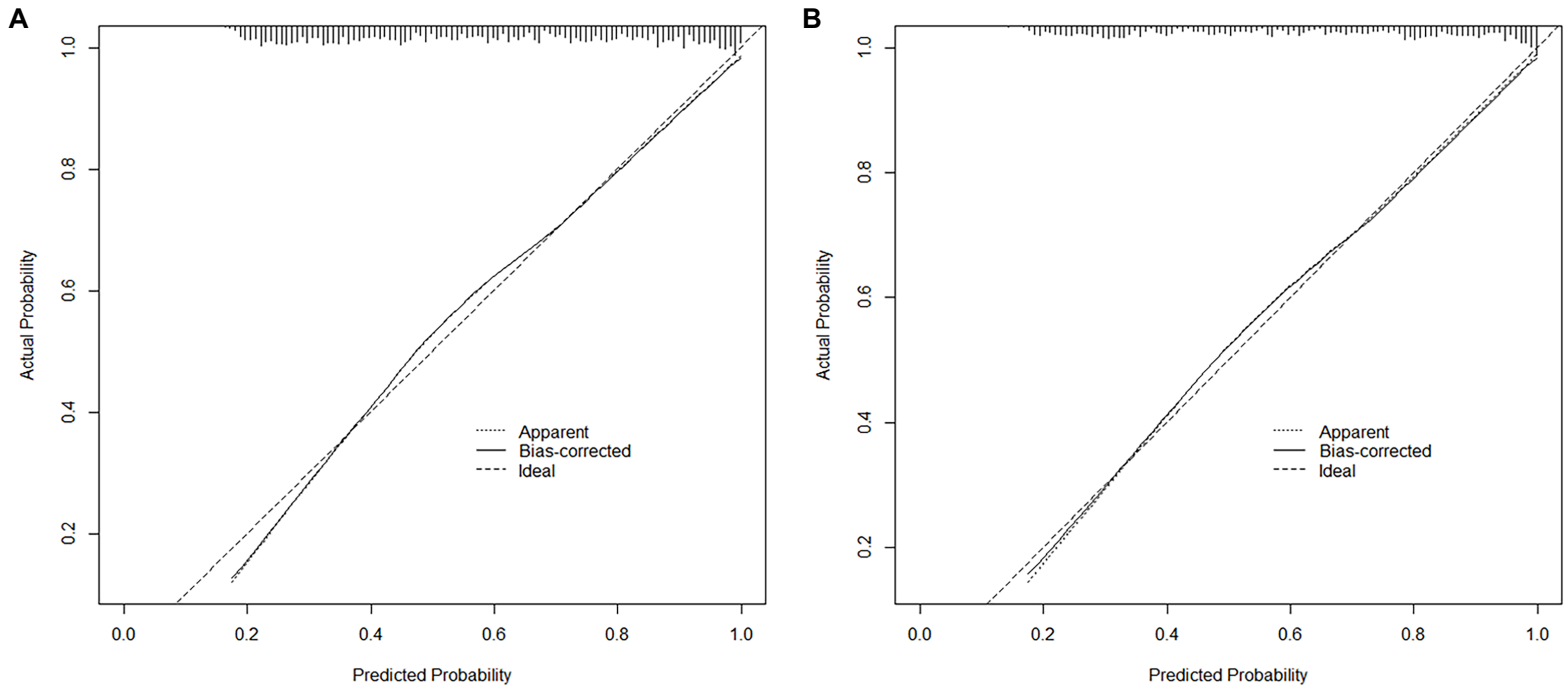
The calibration plots comparing predicted and actual poor sleep quality in the training **(A)** and validation **(B)** cohorts.

**Figure 5:**
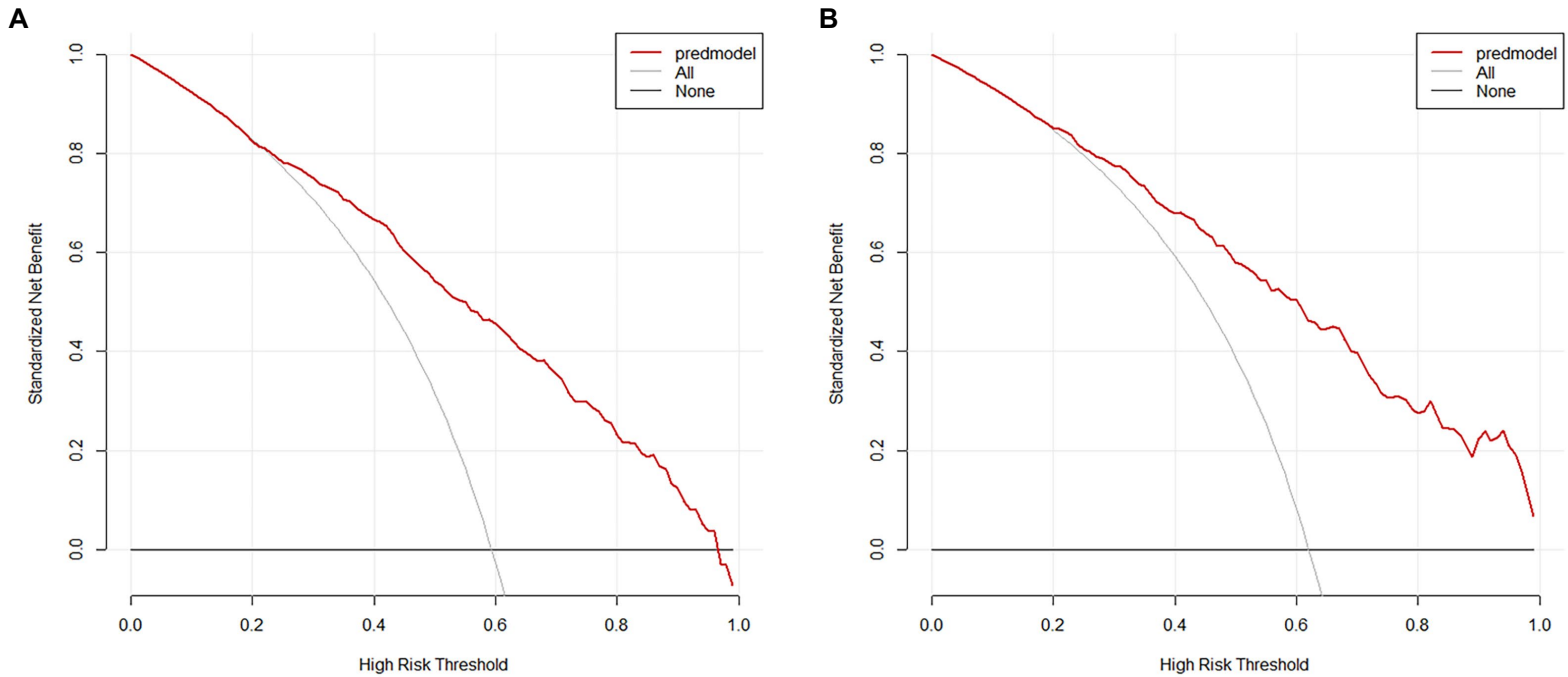
Decision curve analysis of the nomogram in the training **(A)** and validation **(B)** cohorts.

The nomogram developed from the training cohort can be utilized to predict the presence of poor sleep quality, as shown in Figure 2. The probability of poor sleep quality could be evaluated by calculating the total points through a vertical line from the variable to the point axis. The origin and bias-corrected C-index were 0.8075 and 0.8067 in the training cohort, 0.8217 and 0.8190 in the validation cohort, respectively, using 1,000 bootstrap resamples. The areas under the receiver operator characteristic (ROC) curves (AUCs) were 0.808 (95%CI [0.787–0.828]) and 0.822 (95%CI [0.792–0.823]) in the training and validation cohorts, confirming the nomogram’s sufficient predictive ability (Figure 3). ROC curves were further analyzed to determine the optimal cut-off point based on the Youden index in the training and validation cohorts. In the training cohort, the optimal cut-off value for the nomogram was 0.483, exhibiting the best balance between specificity (80.0%) and sensitivity (66.4%). The optimal cut-off value for the nomogram in the validation cohort was 0.658, showing the best balance between specificity (81.9%) and sensitivity (67.9%). Excellent agreement between the predicted and actual incidence of poor sleep quality in both cohorts was shown by the calibration curves with 1,000 bootstrap (Figure 4). Additionally, the decision curve analysis (DCA) curves also demonstrated the excellent clinical utility of the nomogram in the training and validation cohorts (Figure 5).

#### 3.2.4. Predictors for the severity of poor sleep quality

In the zero-truncated negative binomial regression model, only age and PHQ-9 were significant predictors (Table 6), indicating that only age and the degree of depression can predict the severity of poor sleep quality. Each year of age added a factor of 1.004 to the severity of poor sleep quality (RR = 1.004; 95% CI [1.002–1.005]; *p* < 0.001). And predicted severity of poor sleep quality increases by a factor of 1.027 for each unit increase in PHQ-9 (RR = 1.027; 95% CI [1.021–1.032]; *p* < 0.001).

**Table 6:** Risk factors of the severity of poor sleep quality derived from the zero-inflated negative binomial regression analysis.

Characteristics	B (SE)	Risk ratio (95%CI)	*p*-value
Age	0.004 (0.001)	1.004 (1.002,1.005)	<0.001
Gender	0.034(0.024)	1.035 (0.988,1.084)	0.145
Weight	−0.0003 (0.001)	1.000(0.998,1.002)	0.791
Illness duration (5–10 years)	0.007 (0.024)	1.007(0.960,1.056)	0.756
Disease activity	0.001 (0.023)	1.010(0.965,1.057)	0.679
Biologics	0.038 (0.022)	1.039(0.995,1.085)	0.086
PHQ-9	0.026 (0.003)	1.027(1.021,1.032)	<0.001
GAD-7	−0.0001 (0.004)	1.000(0.993,1.007)	0.980
Bowel habits	−0.001 (0.002)	0.999(0.995,1.003)	0.560
Systemic performance	−0.003 (0.004)	0.997(0.990,1.004)	0.365
Emotion performance	0.0003 (0.002)	1.000(0.997,1.004)	0.838
Social performance	−0.001 (0.003)	0.999(0.993,1.005)	0.769

PHQ-9 = Patient Health Questionnaire-9; GAD-7 = Generalized Anxiety Disorder 7-item Scale; OR = odds ratio; CI = confidence interval.

## 4. Discussion

In this national multicenter cross-sectional study, we investigated IBD patients’ clinical and psychological characteristics with 2,478 cases among different age groups. Compared to the CD group (15.36%), the UC group had a higher percentage of elderly patients (39.53%). This is mainly because of the more apparent bimodal age distribution of UC and the fact that the age at diagnosis is 5–10 years earlier for CD than that of UC, as confirmed by prior research in Asia and the West (25, 26). There are substantially more male patients in the younger group of CD patients (73.85%) than in the older group (59.41%). However, such a distribution was not present in UC patients. Male predominance is a characteristic trait of Asian CD patients (27). In Asia-Pacific regions, males have a higher risk of CD from 15 to 50 years old (25). However, Sheila (28) reported that, in Western countries, females had a greater incidence of CD after puberty. The disparities in genetic and environmental factors derived from biological, social, and economic exposures between males and females may account for this variance. Alternately, some studies implicate the action of hormones on the brain-gut-microbiota axis as the source of gender differences in IBD. Nevertheless, this complicated pathophysiology’s mechanism has yet to be fully understood (29).

The incidence and prevalence of IBD among older adults are rising. It is anticipated that over one-third of IBD patients will be over 60 by 2030 (29). Inpatient mortality rates, serious infections, length of stay, and hospital expenses are higher for older patients with IBD. Managing these patients involves unique challenges related to balancing the risk of disease- and treatment-associated complications within the context of overall health. However, evidence-based treatment guidelines for older patients suffering from IBD are lacking (30). We discovered that older patients used biologics less than younger patients, which is consistent with previous observations in other regions (31). This might be due to a milder clinical course or biologics being used less frequently in the elderly, which generates more intensive supervision. However, in our study, there were no significant differences between young and old in terms of disease activity or symptoms. This reflects a cautious attitude among Chinese clinicians regarding the use of biologics in older patients, which might be the main reason. We also found significantly greater rates of corticosteroid use among older patients (18.24%) than younger patients (9.39%) in CD. Being hesitant to use biologics or thiopurines and additional conditions requiring corticosteroids among older patients may result in it. However, previous studies have demonstrated that corticosteroids increased the risk of adverse events in IBD patients above 50 (32), leading to an increased mortality rate in CD patients (33). Therefore, minimizing corticosteroid exposure should be one of the main goals for IBD patients, particularly seniors, to achieve steroid-free remission. And this requires more evidence-based data from researchers on the use of biologics in elderly patients.

PSQI score was the only psychologically relevant variable that significantly differed across age groups with both CD and UC patients. According to our study, 64.89% of older patients with IBD had poor sleep quality, which is much higher than that reported in other studies of healthy individuals (34). It has been long recognized that IBD patients suffer from poor sleep quality. Even asymptomatic patients in remission may experience disrupted sleep, diminishing their quality of life and raising their risk of future IBD flare-ups (35). However, few researchers have analyzed the risk factors of the presence and severity of poor sleep quality in IBD patients (6).

Physiological changes in sleep are normal parts of the ageing process (36). Older IBD patients have a higher incidence of poor sleep quality than younger IBD patients (11). Poor sleep quality is associated with more significant psychological and physical burden in older IBD patients, so it is crucial to tailor sleep management interventions. Due to regional variations in the categorization of age groups and to broaden the applicable population, our study built the hurdle model based on patients across all age groups rather than elderly patients. As a tool for assessing sleep quality, the PSQI score has adequate reliability and validity and is simple to administer. In this study, we included a comprehensive measurement of biopsychosocial variables and a hurdle model that concurrently assessed the presence and severity of poor sleep quality. We did not detect a statistically significant relationship between the two primary forms of IBD and the PSQI score (*p* = 0.737), consistent with previous research indicating no difference in sleep quality between CD and UC patients (6). In our study, IBD activity was not an independent risk factor for poor sleep quality, probably because IBD activity was assessed in the form of a subjective disease activity score, which is consistent with the results of a recent study that showed no significant correlation between subjective activity and sleep quality in IBD patients (11). While old age, high PHQ-9 scores, severe systemic performance and mild emotional performance were found to be significantly associated with the presence of poor sleep quality among IBD patients. It may seem paradoxical that milder emotional performance is associated with poorer sleep quality. In fact, severe emotional performance frequently necessitates more use of psychiatric medication, such as benzodiazepines, benzodiazepine receptor agonists and some antidepressants, which often have a notable impact on improving sleep quality (37).We successfully drew a visualization nomogram using these predictors, which had good performance in predicting the presence of poor sleep quality and a sufficient net benefit for clinical application, as well as excellent discrimination and calibration both in training and validation cohorts. The presence and severity of poor sleep quality were mainly associated with psychosocial factors but not disease factors such as symptoms, disease activity, or therapy type. Age and the PHQ-9 score can be used to predict both the presence and severity of poor sleep quality. Among the IBDQ score, we discovered that systemic and emotional performance were significant variables related to poor sleep quality. The relationship between sleep quality, quality of life, and psychological symptoms is complex. A shared neurological relationship between major depressive disorder and primary insomnia has been shown by studies linking both disorders to deficits in cortical γ-aminobutyric acid and glutamate (38). Over 90% of people with depressive syndromes were reported having sleep problems (39). Psychosocial factors, especially depression, are frequently associated with poor sleep in IBD patients (11). We found that depression was significantly associated with both the presence and severity of poor sleep quality, further confirming the relationship between poor sleep quality and depression in IBD.

The study’s strengths include thorough psychosocial measurement through different questionaries and a hurdle model, which may predict both the presence and severity of poor sleep quality. Additionally, as this is a large, population-based, treatment center-independent study, the results are generalizable. However, there are several limitations to our study. First, we did not collect information on patients’ psychotropic medication use, which became a confounding factor in determining the specific relationship between emotional performance and poor sleep quality. Then, because the study is retrospective and cross-sectional, it is impossible to prove a causal association. Longitudinal studies are required to determine the direction of the relationship between sleep quality, mental health, and quality of life. Furthermore, sleep quality was not objectively measured but estimated by questionnaires, subject to recall bias. Finally, the relevant indicators reflecting objective IBD activity should have been addressed in our study. Future studies should explore the relationship between objective IBD activity-related indicators such as endoscopic and pathological manifestations and sleep quality.

## 5. Conclusion

In conclusion, there were differences in clinical and psychological characteristics between age groups in IBD patients, and the prevalence of poor sleep quality among the older group in IBD patients was relatively high. Age, the PHQ-9 score, and systemic and emotional performance could predict the presence of poor sleep quality. In addition, age and the PHQ-9 score can also predict the severity of poor sleep quality. It suggests that controlling depression may enhance the sleep quality of IBD patients, especially those who are elderly.

## Data availability statement

The raw data supporting the conclusions of this article will be made available by the authors, without undue reservation.

## Ethics statement

The studies involving human participants were reviewed and approved by The Institutional Review Board of Renmin Hospital of Wuhan University. The patients/participants provided their written informed consent to participate in this study.

## Author contributions

SZ, CL, JZ, PA, CJ, JS, KW, and WD: conception and design. WD and KW: administrative support. SZ, CL, JZ, PA, KW, and WD: provision of study materials or patients. SZ, CL, JZ, and PA: collection and assembly of data and data analysis and interpretation. SZ, CL, JZ, PA, ZL, CJ, JS, KW, and WD: manuscript writing. All authors contributed to the article and approved the submitted version.

## Funding

The National Natural Science Foundation of China (No. 82170549) funded this manuscript.

## Conflict of interest

The authors declare that the research was conducted in the absence of any commercial or financial relationships that could be construed as a potential conflict of interest.

## Publisher’s note

All claims expressed in this article are solely those of the authors and do not necessarily represent those of their affiliated organizations, or those of the publisher, the editors and the reviewers. Any product that may be evaluated in this article, or claim that may be made by its manufacturer, is not guaranteed or endorsed by the publisher.
