# Supplementary material for: Risk factors for poor sleep quality in patients with inflammatory bowel disease in China: A multicenter study
Source: Front Psychiatry. 2023 Mar 9;14:1130396. doi: 10.3389/fpsyt.2023.1130396 (PMC10035049; doi:10.3389/fpsyt.2023.1130396)
Supplement: Supplementary file 1 [file Data_Sheet_1.docx]

Supplementary Material

Risk Factors for Poor Sleep Quality in Patients with Inflammatory Bowel Disease in China: A Multicenter Study

Suqi Zeng *, Chuan Liu, Jixiang Zhang, Ping An, Zhongchun Liu, Changqing Jiang, Jie Shi, Kaichun Wu, Weiguo Dong, Psychology Club of Inflammatory Bowel Disease Group, Chinese Society of Gastroenterology, Chinese Medical Association, Chinese Association for Mental Hygiene

*** Correspondence:**

Weiguo Dong*, [dongweiguo@whu.edu.cn](mailto:dongweiguo@whu.edu.cn)

Kaichun Wu*,[kaicwu@fmmu.edu.cn](mailto:kaicwu@fmmu.edu.cn),

# Supplementary Figures and Tables

## Supplementary Figures


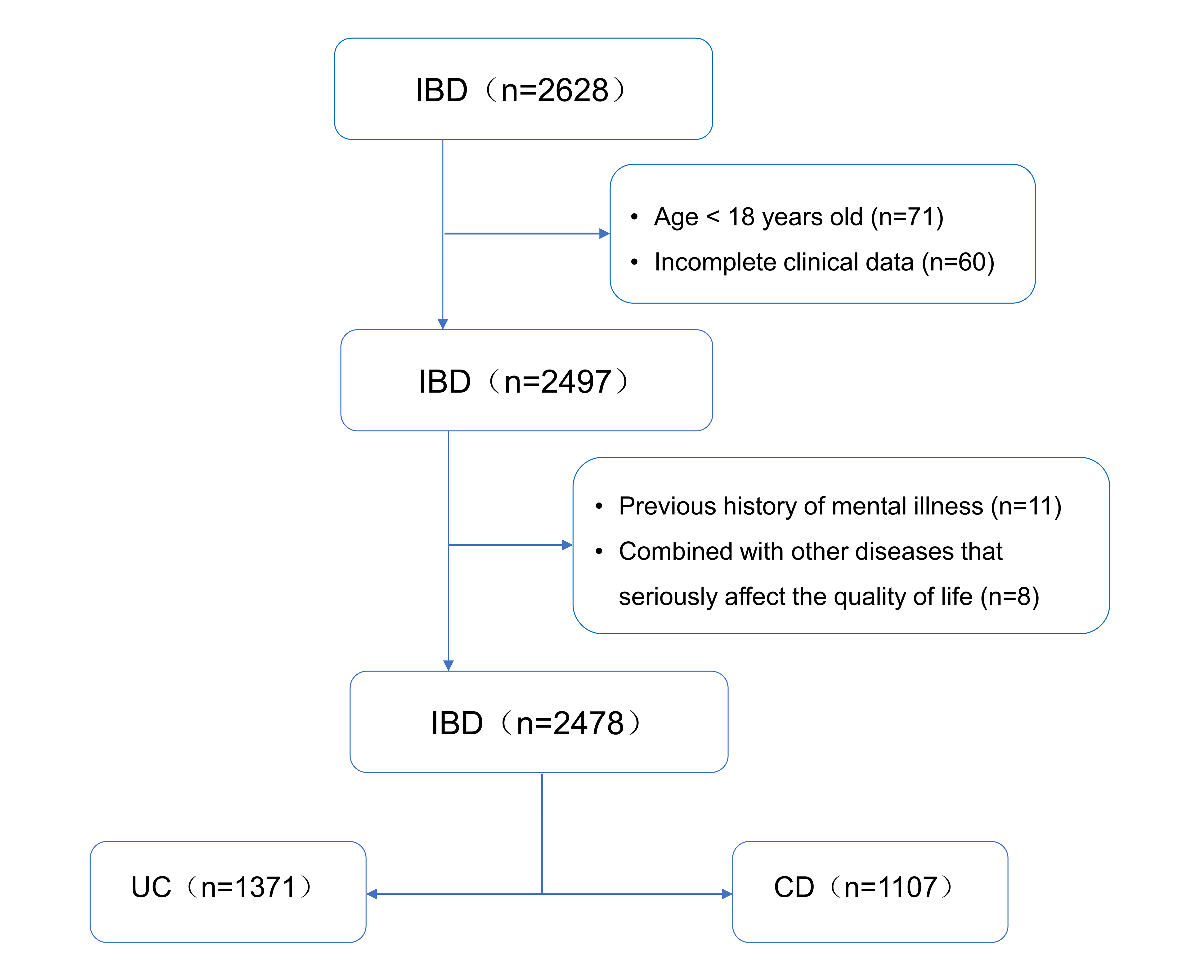


**Supplementary Figure 1.** Flow chart of the subject selection process. (CD, Crohn's disease; UC, ulcerative colitis; IBD, inflammatory bowel disease)


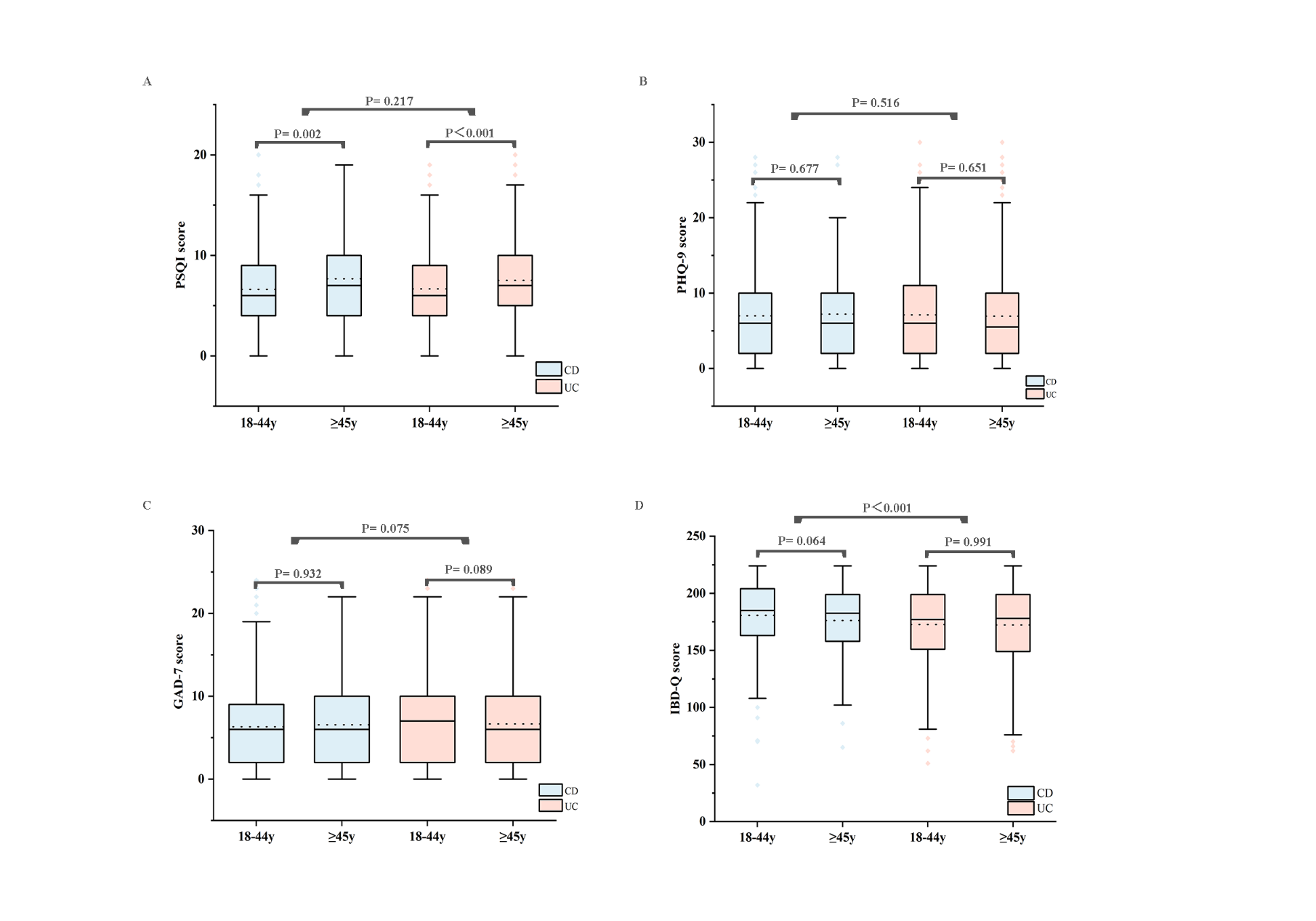


**Supplementary Figure 2.** PSQI (A), PHQ-9 (B), GAD-7 (C) and IBDQ scores (D) in IBD patients of different age groups. (CD, Crohn's disease; UC, ulcerative colitis; PSQI, Pittsburgh Sleep Quality Index; PHQ-9, Patient Health Questionnaire-9; GAD-7, Generalized Anxiety Disorder 7-item Scale; IBD-Q, Inflammatory Bowel Disease Quality-of-Life Questionnaire)


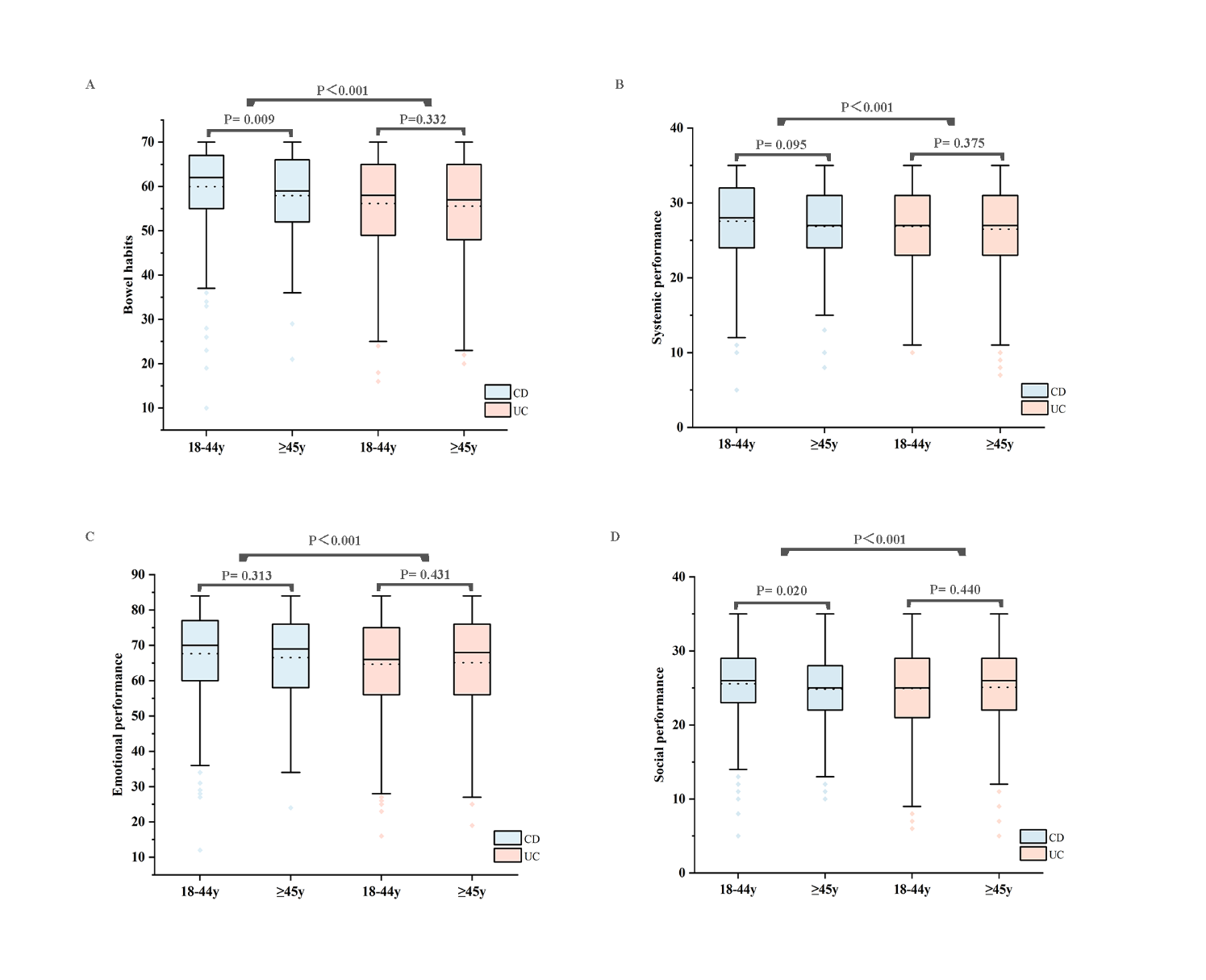


**Supplementary Figure 3.** The scores of bowel habits (A), systemic performance (B), emotional performance (C) and social performance (D) in CD and UC patients of different age groups. (CD, Crohn's disease; UC, ulcerative colitis; IBD, inflammatory bowel disease)


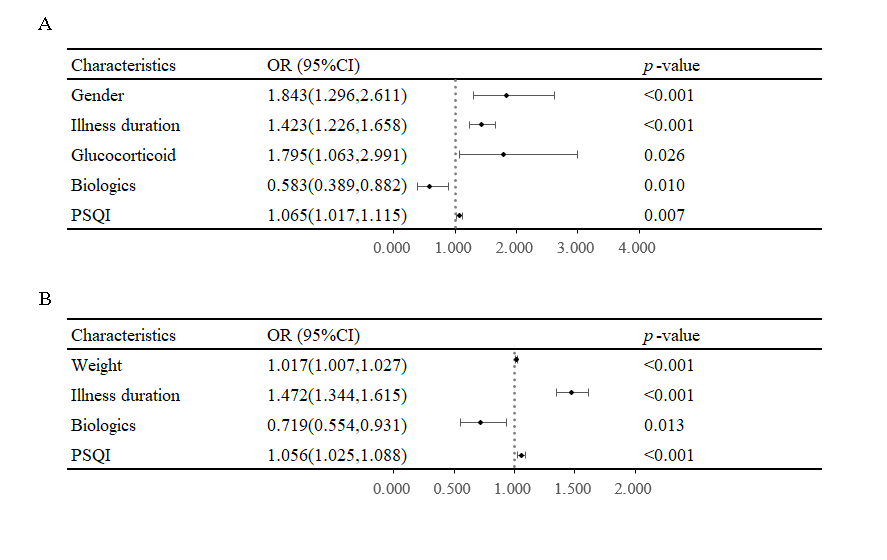


**Supplementary Figure 4.** Multivariate analysis of factors associated with age in CD (A) and UC (B) patients. (CD, Crohn's disease; UC, ulcerative colitis; PSQI, Pittsburgh Sleep Quality Index; OR, odds ratio; CI, confidence interval)

## Supplementary Tables

|  | CD | UC |  |
| --- | --- | --- | --- |
|  | N=1107(44.67%) | N=1371(55.33%) | *P* |
| PSQI | 6.77±3.71 | 7.00±3.81 | 0.217 |
| PHQ-9 | 7.02±5.91 | 7.05±6.26 | 0.516 |
| GAD-7 | 6.35±4.93 | 6.87±5.47 | 0.075 |
| IBDQ | 180.00±28.41 | 172.44±32.57 | <0.001 |
| Bowel habits | 59.63±8.95 | 55.92±10.83 | <0.001 |
| Systemic performance | 27.44±5.15 | 26.70±5.60 | <0.001 |
| Emotional performance | 67.48±12.15 | 64.84±13.14 | <0.001 |
| Social performance | 25.45±4.78 | 24.99±5.21 | 0.046 |

**Supplementary Table 1.** PSQI, PHQ-9, GAD-7 and IBDQ scores in CD and UC patients. (CD, Crohn’s disease; UC, ulcerative colitis; PSQI, Pittsburgh Sleep Quality Index; PHQ-9, Patient Health Questionnaire-9; GAD-7, Generalized Anxiety Disorder 7-item Scale; IBDQ, Inflammatory Bowel Disease Quality-of-Life Questionnaire)

|  | 18-44y | ≥45y |  | |  |
| --- | --- | --- | --- | --- | --- |
|  | N=1766(71.27%) | N=712(28.73%) | χ2 | *P* | |
| Depression | 530(30.01%) | 207(29.07%) | 0.17 | | 0.679 |
| Anxiety | 444(25.14%) | 46(26.26%) | 0.28 | | 0.597 |
| Poor sleep quality | 1029(58.27%) | 462(64.89%) | 9.01 | | 0.003 |

**Supplementary Table 2.** The proportion of depression, anxiety, and poor sleep quality in IBD patients of different age groups. (IBD, inflammatory bowel disease)
